# MRI Volumetric Changes in Perianal Fistulizing Crohn's Disease: Moving Toward a Novel Outcome Measure for Therapeutic Response

**DOI:** 10.1002/ueg2.12765

**Published:** 2025-01-30

**Authors:** Raja Atreya, Markus F. Neurath

**Affiliations:** ^1^ First Department of Medicine Erlangen University Hospital Friedrich‐Alexander‐Universität Erlangen‐Nürnberg Erlangen Germany; ^2^ Deutsches Zentrum Immuntherapie (DZI) Erlangen Germany

Crohn's disease is a chronic, immune‐mediated disorder of the gastrointestinal tract, that is characterized by a relapsing‐remitting course with heterogeneous phenotypes. Perianal fistulizing Crohn's disease represents one of the most aggressive and debilitating forms of Crohn's disease, characterized by abnormal connections between the rectum or anal canal and adjacent tissue, occurring with an estimated prevalence of 14%–43%. The impact of this condition can be detrimental to a patient's quality of life, with increased severity of symptoms and an overall poor prognosis. The management of perianal fistulizing Crohn's disease is a major clinical challenge due to its refractoriness to medical treatment despite recent advancement in targeted therapies, as many patients still require surgical intervention [1].

Detailed evaluation of the fistula tract is essential to determine the optimal management strategy and to assess responsiveness to initiated therapies. Here, magnetic resonance imaging has established itself as the reference standard to assess the anatomy, the degree of inflammation and treatment response in Crohn's disease associated fistula tracts [2]. Correspondingly, radiological healing was associated with fewer symptoms than clinical healing alone and also predicted persistent fistula closure [3]. Therefore, clinical trials have lately used combined clinical and radiologic outcome measures as a combined primary endpoint [4]. However, it still remains unclear which imaging features demonstrate a healed fistula, where the threshold for determining therapeutic response lies and how to define fistula healing [5]. There is an unmet need to define a radiologically healed fistula on MRI, that would correlate with beneficiary clinical outcomes and could be used in clinical practice, as well as in clinical trials.

In the current issue of the *United European Gastroenterology Journal*, Caballol and colleagues investigated changes in 3D volumetry of perianal fistulas in patients with Crohn's disease via pelvic MRI in an observational unicentric and retrospective study. 24 patients with active and predominantly complex perianal fistulas treated with biological therapy were included in pre‐ and post‐treatment (3–18 months) MRI assessment and subsequent clinical follow‐up of 2–5 years. Applying a novel software method, 3D volumina of the fistulas and their active and fibrotic components were evaluated. The active component was defined as the sections presenting hypersignal in the T2‐weighted sequences with fat suppression, interpreted as either fluid or granulation tissue, while the fibrotic component was defined as the area presenting hyposignal in T2‐weighted sequences with and without fat suppression. Following the manual segmentation of each component, a software program automatically generated a three‐dimensional model and the volume of each component, whether active or fibrotic, was obtained by summing the area in each section. Logistic regression analysis identified relative changes in the volumetric active component of the fistula as an independent predictor of clinical remission, which was defined as absence of external opening fistula drainage. A reduction of ≥ 16% in the volumetric percentage of the active component had a sensitivity of 84.6% and specificity of 81% in predicting clinical remission during follow‐up. These findings demonstrate the potential value of this novel diagnostic method for assessing therapeutic response in Crohn's disease perianal fistula based on reduction in the volumetry of the active component.

Altogether, this is a convincing pilot study addressing a currently unmet need in Crohn's disease, as objective evaluation of fistula tract response to initiated therapies still remains a challenging task. Various studies have indicated that fistula healing identified by MRI is a valuable outcome measure, as it is associated with prolonged period of clinical remission [6]. There is currently no consensus definition of a radiologically healed fistula on MRI in perianal fistulizing Crohn's disease, but it is usually based on the reduction of the active and a consecutive increase in the fibrotic component. Analysis of the PISA‐II study also indicated, that fibrosis of the fistula tract is associated with long‐term clinical closure and can therefore be utilized to define radiological healing [7]. Results of the presented volumetric MRI study fittingly suggests that a reduction in the volume of the active component of the fistula tract predicts subsequent clinical remission in treated patients and could therefore be used as a predictive marker. These findings indicate, that further novel imaging methods like Ga‐68‐FAPI‐46 and F‐18‐FDG‐PET/CT imaging, which has successfully been applied to distinguish inflammation from fibrosis in strictures of patients with Crohn's disease patients, could also be utilized to characterize perianal fistulas [8].

Some limitations of the presented study need to be acknowledged, which include the retrospective study design, the unicentric setting, the small sample size of included patients and the limited follow‐up time. Further data regarding the timing of observed volumetric changes between initiated therapies and differing outcomes concerning the type of treated perianal fistula would provide valuable further insights for prospective validation of this novel diagnostic procedure. Furthermore, differentiation of fibrotic and active components based on signal intensity on T2 sequences should be validated against histological analyses of the investigated fistula tract. This diagnostic method should ideally also include outcomes for both perianal and internal fistulae. Reduction in the volume of the active component of the fistula should also be correlated with newly proposed patient‐reported outcome measures, such as the Crohn's Anal Fistula Quality of Life scale [9].

Nevertheless, this study is of great value compared to the available literature, notably by being the first to assess volumetric changes of active and fibrotic components of the fistula tact and positively correlating it with clinical response to therapy. Overall, decrease in the active component in 3D volumetry on MRI might represent a valuable biomarker for defining therapeutic targets of treatment of perianal fistulizing disease, thereby addressing an unmet need in clinical practice and trials of patients with Crohn's disease [10].

## Conflicts of Interest

R.A. has served as a speaker, or consultant, or received research grants from AbbVie, Abivax, Astra‐Zeneca, Bristol‐Myers Squibb, Celltrion Healthcare, Galapagos, Johnson&Johnson, Lilly, MSD, Pfizer, and Takeda Pharma. DFG‐SFB/TRR241 Project IBDome, KFO5024 B04 are funded by the German Research Council (DFG). M.F.N. has served as advisor or speaker for Pentax, Roche Pharma, Takeda Pharma, Pfizer, MSD, PPM, Janssen, Gilead, Dr Falk Pharma, Boehringer Ingelheim, Amgen and AbbVie.

## Data Availability

Data sharing is not applicable to this article as no new data were created or analyzed in this study.
